# Association between Six Environmental Chemicals and Lung Cancer Incidence in the United States

**DOI:** 10.1155/2011/463701

**Published:** 2011-07-10

**Authors:** Juhua Luo, Michael Hendryx, Alan Ducatman

**Affiliations:** ^1^Department of Community Medicine, School of Medicine, West Virginia University, P.O. Box 9190, Morgantown, WV 26506, USA; ^2^West Virginia Rural Health Research Center, West Virginia University, P.O. Box 9190, Morgantown, WV 26506, USA; ^3^Mary Babb Randolph Cancer Center, West Virginia University, P.O. Box 9190, Morgantown, WV 26506, USA

## Abstract

*Background.* An increased risk of lung cancer has been observed at exposure to certain industrial chemicals in occupational settings; however, less is known about their carcinogenic potential to the general population when those agents are released into the environment. *Methods.* We used the Toxics Release Inventory (TRI) database and Surveillance, Epidemiology, and End Results (SEER) data to conduct an ecological study at the county level. We used multiple linear regression to assess the association of age-adjusted lung cancer incidence with the quantities of on-site air and water releases of six selected industrial chemicals including arsenic, 1,3 butadiene, cadmium, chromium, formaldehyde, and nickel after controlling for other risk variables. *Results.* Overall, we observed a significantly increased risk of lung cancer incidence associated with releases of chromium, formaldehyde, and nickel. The links were present for both males and females. Significant effects were present in nonmetropolitan but not metropolitan counties. Releases of arsenic, 1,3 butadiene, and cadmium were reported by small numbers of facilities, and no relationships to lung cancer incidence were detected. *Conclusions.* Our results suggest that environmental exposure to chromium, formaldehyde, and nickel from TRI sites may increase population risk of lung cancer. These findings need to be confirmed in individual-level studies, but in congruence with the precautionary principle in environmental science, support prudent efforts to limit release of these agents into the environment.

## 1. Introduction

Lung cancer is the leading cause of cancer-related death, responsible for over one million deaths worldwide each year [1]. Smoking is the most commonly identified risk factor for lung cancer. However, 10–15% of all patients with lung cancer worldwide do not report smoking tobacco over their life time [2, 3]. Risk factors that have been identified for lung cancer in people who have never smoked [2] include second-hand exposure to tobacco smoke [4, 5], radon [6, 7], indoor air pollution including pollutants generated by combustion of coal and biomass in the household [8], and some occupational agents, including asbestos [9]. However, a large fraction of lung cancers occurring in never-smokers remain in the absence of clear environmental risk factors. 

Several heavy metals including arsenic, cadmium, chromium, and nickel have been implicated in the increased risk of lung cancer [2]. Other than arsenic, evidence for metals causing lung cancer outside of workplaces has been inconsistent [10–15]. Epidemiological studies have also suggested that occupational exposure to formaldehyde, or to 1,3-butadiene may increase risk of lung cancer [16, 17]. However, again, results have been inconsistent [18–23]. A majority of the studies on the carcinogenicity of these agents have been conducted in highly exposed occupational groups, or in some populations with unusual exposures. Little is known about the carcinogenic potential of those agents in general population settings, which usually entail lower levels of exposure than those seen in occupational settings. Considering the time lag between exposure and the development of illness, assessing past exposure to environmental pollution is another factor that increases the difficulty in studying this question in the general population. 

Previous studies have demonstrated that environmental pollutants including those originating from hazardous waste sites, industrial emissions, or agricultural pesticide use increase risk of adverse health outcomes in humans [24–29]. However, only a few studies have examined the effects of specific environmental toxicants on the risk of lung cancer [26, 27]. In this study, we used the Toxics Release Inventory (TRI) database and Surveillance, Epidemiology, and End Results (SEER) data to conduct an exploratory, ecological study to assess the association of releases of six selected industrial chemicals with lung cancer incidence at the county level in the USA. Those chemicals include arsenic, 1,3-butadiene, cadmium, chromium, formaldehyde, and nickel, which we selected based on their possible impact on lung cancer risk as suggested by prior literature. All six selected agents were TRI-reported chemicals that met the Occupational Safety and Health Administration (OSHA) carcinogen standard and have been classified as a group 1 carcinogen by the IARC [30]. Organic arsenic compounds are not classified as group 1 carcinogens and so were not included in the study.

## 2. Methods

### 2.1. TRI Database

The Toxics Releases Inventory (TRI) database is considered to be the most comprehensive data source on industrial toxic emissions in the USA [31]. The TRI database was originally established under the Emergency Planning and Community Right-to-Know Act (EPCRA) in 1986 [32]. EPCRA requires manufacturing facilities that meet certain thresholds (have 10 or more full-time employees and manufacture or process over 25,000 pounds annually or otherwise use more than 10,000 pounds of any chemical specified on the TRI list) to annually report their estimated releases and transfers of toxic chemicals to the U.S. Environmental Protection Agency (EPA). Releases include unplanned spills and routine emissions of chemicals released directly to the air and land, injected into land, discharged to surface water, or transferred to publicly owned treatment works commonly known as sewage treatment plants or other off-site locations for recycling and waste disposal. Failure to report can result in civil penalties, monetary payments of the economic benefits of noncompliance, and required correction of the violation. Suspected violations may be reported to the EPA from government agencies, organizations, or individual citizens. However, the system relies on measurements conducted by the facilities themselves and on voluntary reporting by facilities. 

The TRI database is designed to encourage pollution prevention and waste reduction by increasing public access and knowledge of environmental chemical releases. However, this environmental information resource has been underexploited for research purposes.

### 2.2. Measurements of Exposure

The TRI database is available online with data on chemical releases beginning in 1987. In this study, we used the TRI database to extract total TRI on-site releases for six selected chemicals from 1988 through 1990. We excluded 1987 data due to concerns that the dataset was still developmental and incomplete in its first year. On-site releases include those to air, water, surface land, and surface injections. The data were downloaded from the EPA website [33]. For each selected chemical, we calculated the average annual release between 1988 and 1990 at the county level, measured in pounds. The amount of toxicant release for each selected chemical was found to be non-normally distributed, and therefore we conducted a natural logarithm transformation for the analysis. Each selected chemical was also classified as a dichotomous variable (zero release or nonzero release) without regard to the amount of release. 

In addition, we derived two variables based on the total amount of release summed across all selected chemicals combined. First, the total amount of release for all selected chemicals was divided into low or high release using a median cut point of 2000 annual pounds resulting in a similar number of counties in each group (low release: <2000 pounds, high release: ≥2000 pounds). Second, the total amount of release of all selected chemicals was summed and subject to a natural logarithm transformation in the analysis. As a final exploratory measure, we created a measure that was the sum of TRI sites per county regardless of the level or type of release.

### 2.3. SEER Database

The Surveillance, Epidemiology, and End Results (SEER) Program of the National Cancer Institute (NCI) is an authoritative source of information on cancer incidence and survival in the USA [34]. SEER currently collects and publishes cancer incidence and survival data from 17 population-based cancer registries covering approximately 26 percent of the US population [34].

In this study, we extracted data on age-adjusted lung cancer incidence rates between 1992 and 2007 from 13 SEER registries using the SEER∗Stat Software developed by the SEER Program of the National Cancer Institute [35]. We used all 13 registries that were participating in the SEER program prior to the year 2000. The 13 SEER registries are Atlanta, Connecticut, Detroit, Hawaii, Iowa, New Mexico, San Francisco-Oakland, Seattle-Puget Sound, Utah, Los Angeles, San Jose-Monterey, Rural Georgia, and Alaska, including a total of 225 counties. After linking with the TRI dataset, 215 counties remained for analyses. 

In addition, county-attributed data for 1990 were extracted with the same software (SEER∗Stat) to gather data on potential confounders, including proportion of nonwhite population, proportion of male population, proportion of people with less than high school education, proportion of people with college or higher education, proportion of families below poverty, proportion unemployed, and rural-urban continuum codes. The 1993 US Department of Agriculture Rural-Urban Continuum Codes 0–3 were defined as metro counties, and Rural-Urban Continuum Codes 4–9 were defined as nonmetro counties. The county attribute variables for 1990 are calculated using the 1990 Census TF1 (Sample Tape File 1) and TF3 (Sample Tape File 3) files. Prevalence of smoking for each county was obtained from Behavioral Risk Factor Surveillance System (BRFSS) data based on samples conducted in 2003 and 2006, supplemented with additional county smoking rate estimates based on review of state public health department websites. 

After consideration of the time period covered by the various databases (TRI data are available to 1988, the 13 SEER registries are available beginning in 1992, and the US census data is available in 1990, which matches the other database periods better than the 2000 census), we limited information on exposures and confounders up to 1990 and examined their effects on lung cancer incidence between 1992 and 2007 in order to ensure that exposures preceded disease.

### 2.4. Statistical Analysis

Age-adjusted rates of lung cancer incidence from the SEER data were linked with TRI chemical releases at the county level. The characteristics of the 215 counties were compared first among different levels of release of the selected chemicals (zero, low, and high) using Chi-square tests for categorical variables and ANOVA tests for continuous variables. 

Univariate and multivariate linear regression analyses were used to determine the association of age-adjusted lung cancer incidence with these environmental toxicants. Prior to the regression analyses, we examined the bivariate Pearson correlations among the covariates for collinearity, and excluded two variables, unemployment rate and percent of the population without a high school education, because they correlated highly with poverty rate and with each other. In the multivariate analyses, after taking collinearity into account, we adjusted for potential confounders including proportion of nonwhite population, proportion of male population, proportion of people with college or higher education, proportion of families below poverty, and metro or nonmetro county. Further, we assessed the association of age-adjusted lung cancer incidence with selected chemicals stratified on gender and on metropolitan or nonmetropolitan counties. For selected significant results, we also estimated relative risks by log transformation of the dependent variable and then exponentiating back the resulting coefficients.

Statistical Analysis Software (SAS) version 9.1 was used for all analyses.

## 3. Results

Table 1 presents 1990 sociodemographic characteristics for the 215 counties grouped by release levels for the selected chemicals. Counties with high-release amounts of the selected chemicals had higher proportions of minority populations, although the difference did not reach statistical significance due to wide dispersion of the proportions of minority populations ranging from 0 to 80%. Other characteristics in the high release group included higher college education rates and smaller proportion of families below poverty. There were no significant differences in unemployment rate and in the prevalence of smoking between counties with different release amounts. The proportion of male population was significantly different across groups due to the small variability in this variable across counties. Counties with no releases were more likely to be located in nonmetropolitan areas, and counties with high release levels were more likely to be in metropolitan areas (Table 1).

The association of each selected chemical with the risk of lung cancer incidence is presented in Table 2. In the unadjusted analyses, we observed a significantly increased risk of lung cancer incidence associated with nonzero releases of chromium, formaldehyde, and nickel, and with high release of all selected chemicals combined. After adjusting for potential confounders in the multivariate analyses, the strength of the association for each chemical became weaker. However, the link for chromium, formaldehyde, and nickel remained significant. In addition, we observed a significant dose-response relationship of lung cancer incidence with chromium, formaldehyde, nickel and the total amount of release for all selected chemicals based on analyzing the log-transformed amount of chemical release as a continuous variable in the model (Table 2). Among the chemicals selected for study, formaldehyde showed the strongest association with lung cancer incidence. After adjusting for potential confounders, the counties with any formaldehyde release had an excess age-adjusted lung cancer incidence rate of 9.08/100,000 compared to counties without formaldehyde release. The counties with high release of all selected chemicals (over 2000 pounds of total amount) had 12.34/100,000 higher age-adjusted lung incidence compared to counties without release of any selected chemicals. The relative risk (RR) and 95% confidence intervals (CIs) for these two findings are RR = 1.14 (CI = 1.05, 1.24) for formaldehyde release (yes versus no) and RR = 1.21 (CI = 1.11, 1.32) for high total release relative to areas with zero release. We did not observe significant associations for releases of arsenic, 1,3-butadiene, and cadmium, and note that the number of counties with nonzero releases of these chemicals was 2, 12, and 11 counties, respectively (Table 2).

Analyses stratified on gender revealed that the associations between releases of chemicals and lung cancer incidence were similar to the main analyses for both males and females (Table 3). When analyzing the data stratified by metro or nonmetro counties, we observed that the increased risk of lung cancer incidence associated with chromium, formaldehyde, and nickel was present among nonmetro counties, but not among metro counties (Table 4). The RR values for the two most significant Table 4 findings were 1.18 (CI = 1.05, 1.33) for formaldehyde releases (yes versus no) in nonmetro counties and 1.29 (CI = 1.13, 1.46) for high total release relative to areas with zero release.

As a final linear regression analysis, we used as the primary independent variable the number of TRI sites per county, rather than the releases of the six chemicals. Controlling for the same covariates, we found that the number of sites was significantly associated with greater lung cancer incidence, overall, for both genders, and in nonmetropolitan but not metropolitan counties. However, when we added the released amounts of the chemicals to the models, the released amounts were significantly related to lung cancer incidence in the same way as reported in Tables 2–4, but the number of sites was no longer significant (results not shown).

## 4. Discussion

We observed a significantly increased risk of lung cancer incidence associated with both nonzero and high volume TRI release of chromium, formaldehyde, and nickel, but not releases of arsenic, 1,3-butadiene, or cadmium. These associations were present within nonmetropolitan counties but not metropolitan counties. 

Our positive finding for chromium release is consistent with most previous observations in occupational settings that have consistently shown excess risk for lung cancer among workers in chromate production, chromate pigment production, and chrome plating [36, 37]. Chromium is a ubiquitous environmental and industrial contaminant. It occurs in the environment primarily in two valence states, trivalent chromium (Cr III) and hexavalent chromium (Cr VI). Cr VI, which is more toxic than Cr III, is most commonly produced by industrial processes including those from mining, chemical processing, metal plating and alloy manufacture, cement plants, and leather and textile manufacturing. Our data are indirect and not definitive, but our findings suggest that, although the highest levels of chromium exposure occur in industrial settings and are carcinogenic to workers, chromium exposure at lower levels in community settings may also be carcinogenic to the general population. 

Formaldehyde is a ubiquitous indoor air pollutant that is used in building materials and to produce household products. Formaldehyde is also commonly used as an industrial fungicide, germicide, and disinfectant and as a preservative in medical laboratories. In 1980, laboratory studies showed that exposure to formaldehyde could cause nasal cancer in rats [38], and since then, the carcinogenicity of formaldehyde in humans has become a major concern. To date, three large cohort studies of cancer risk among workers exposed to formaldehyde have been conducted [16, 18, 22]. One UK study suggested increased risk for lung cancer [16]; two other studies did not observe an increased risk of lung cancer associated with formaldehyde exposure [18, 22]. Our findings are consistent with the concern that exposure to environmental formaldehyde may increase the risk of lung cancer incidence in the general population. Additional studies with stronger designs and exposure assessments are needed to confirm the ecological association observed in our study. 

Overall, we observed an increased risk of lung cancer associated with greater amounts of nickel release. In 1990, a working group of the International Agency for Research on Cancer evaluated epidemiologic and experimental studies of nickel-related cancer, mainly concerning lung and nasal cancer, and concluded that nickel compounds were carcinogenic to humans [39]. More recent epidemiological studies also observed that occupational exposure to nickel is associated with a higher incidence of human lung cancer [12, 40], although such findings are not universal [41]. 

Arsenic is a well-known environmental toxicant and carcinogen. An increase in the incidence of lung cancer at high arsenic concentrations is well established [28, 29, 42, 43], although not all studies have observed this [44, 45]. Similarly, cadmium is a long recognized lung carcinogen [46]. Human exposure to these metals is common because of their widespread use in industry and their environmental persistence. The metallic carcinogenicity is generally thought to generate free radicals, which may play a role in lung tumorigenesis [37]. However, epidemiological studies for cadmium have been inconsistent [47–50]. Our study did not observe a significant excess risk of lung cancer associated with arsenic and cadmium. The inconsistencies may be due to different exposure levels among different studies. Given that increased risks have almost exclusively been observed at high levels of exposure to those agents in occupational settings, it is likely that the exposure levels of those agents in the general population were insufficient to produce observable effects. There were only a few counties with nonzero release of arsenic (*N* = 2) or cadmium (*N* = 12) in our study; thus, low statistical power may be a possible explanation for the null findings. 

1,3-butadiene has been classified as group 1 carcinogen by the IARC. The epidemiological evidence supporting this classification comes mainly from studies of leukemia and non-Hodgkins' lymphoma among male workers exposed to 1,3-butadiene [19]. Despite the recognition of 1,3-butadiene as a potent carcinogen in mice, our results and findings from epidemiological studies of 1,3-butadiene-exposed works [19, 51, 52] support that 1,3-butadiene exposure may not appreciably increase lung cancer risk. However, as with cadmium and arsenic, there were few (*N* = 11) counties with documented releases of this chemical, so we may have little statistical power to detect effects that may be present.

In addition, we observed that the increased risk of lung cancer incidence associated with chromium, formaldehyde, and nickel appeared only among nonmetropolitan counties, but not metropolitan counties. Reasons for the difference are unclear. One possibility may be larger exposure misclassification in metropolitan counties. That is, the stronger associations for nonmetropolitan areas may be due to the presence of many other risk factors present in metropolitan areas from transportation or other point pollution sources that might overwhelm the effects of TRI releases. Our model assumes that all people in a county were exposed to the same quantity of chemicals because of the nature of the ecological design. This assumption may lead to nondifferential exposure misclassification, rendering observed effects in metropolitan areas more conservative toward the null. 

Limitations of this study primarily relate to the nature of the ecological design. We did not have environmental toxicant data pertinent to each individual. Moreover, we were unable to adjust for occupational exposure, smoking habits, and other individual risk factors. In addition, we only had smoking information at the county level during 2003 and 2006, which may not reflect variability in the prevalence of smoking between counties during the same period of time covered by other covariates. The assumption that all people in a county were exposed to the same quantity of chemicals is a simplification, especially in a large county. We did, however, observe that the quantity of chemical release was more important than the number of facilities, which suggests that the three chemicals are themselves important rather than the effect being the consequence of unmeasured confounders pertaining to where TRI sites are located. 

Other limitations pertain to the use of TRI data and to temporal imperfections in the data. TRI data only apply to facilities that emit large volumes of specific contaminants, and they count only emissions from facilities with at least 10 employees that manufacture or process in excess of 25,000 pounds of a listed chemical annually or use an excess of 10,000 pounds annually. Smaller facilities, and those that manufacture or use lesser amounts of a chemical annually, do not report, but may release, substantial levels of pollutants as well. In addition, the accuracy of reported quantities of chemical releases in the TRI may be a concern. There is evidence that some facilities underreport TRI emissions, resulting in systematic measurement errors in the database [53]. Further, we assumed emissions from one county make a negligible contribution to the exposure conditions in neighboring counties. In reality, a facility located near the border between two counties may well contribute equally to the exposure of people in both counties. Thus, studies incorporating spatial analysis methods could contribute to a better understanding of population effects. Another concern is that the exposure time to environmental carcinogens that are necessary for the development of lung cancer is unknown. Our study design does not permit conclusions about the temporal relation between chemical exposure and lung cancer, although we did measure releases occurring over an interval of time prior to the time interval used to capture cancer incidence. By choosing to examine TRI releases occurring prior to disease incidence, the analysis precludes examination of possible “late-stage” carcinogenic effects where exposure and disease occur over a short timeframe, but our approach offers a more conservative assessment of the longer time periods that occur between exposure and disease for most lung cancer cases. We did not address the duration of exposure to chemical releases, nor the possible impacts of county in- or outmigration, although migration would tend to render observed effects conservative. Finally, carcinogens including formaldehyde and cadmium present in cigarette smoke [54, 55] are considered, but only at the county level.

## 5. Conclusions

In conclusion, our study employs national data at the county level and so requires additional confirmatory work. However, our results suggest that environmental exposure to chromium, formaldehyde, and nickel may be important determinants in lung cancer development. These findings stress the need for additional efforts to study public exposures at the individual level. Since our findings concern known carcinogens, the precautionary principle in environmental science, similar to the medical dictum to “do no harm”, would suggest that it is prudent to take reasonable steps to limit release of these agents into the environment.

##  Conflict of Interests

The authors declare that they have no Conflict of interests.

##  Authors' Contributions

J. Luo conceived the study, prepared the data, conducted the analysis, and drafted the manuscript. M. Hendryx contributed to study design, data analysis, data interpretation, and writing. A. Ducatman contributed to data interpretation and drafting the manuscript. All authors read and approved the final manuscript.

## Figures and Tables

**Table 1:** Characteristics of 215 counties by levels of release of selected chemicals*.

Variable^†^	No release	Low release	High release	*P* value
*N* of counties	147	38	30	NA
Proportion of nonwhite population (%)	7.7	9.4	12.8	0.3
Proportion of males (%)	49.0	48.9	49.8	0.004
Less than high school (%)	24.5	22.3	20.3	0.03
College or higher education (%)	14.3	16.9	22.4	<0.0001
Families below poverty (%)	11.9	9.6	8.3	0.005
Unemployed rate (%)	5.7	5.4	6.1	0.6
Prevalence of smoking	18.5	18.1	18.0	0.8
Nonmetro counties (%)^‡^	130 (88.4%)	26 (68.4)	9 (26.7)	<0.0001

*The cut point for low or high release of total amount of selected chemicals was set at 2000 pounds annually (low: <2000 pounds, high: ≥2000 pounds). ^†^All county attributes were in year 1990 except for prevalence of smoking, which was in year 2003–2006. ^‡^Metro or nonmetro counties (metro counties: Rural-Urban Continuum Code < 4; nonmetro counties: Rural-Urban Continuum Code ≥ 4).

**Table 2:** Association of lung cancer incidence with six selected chemicals using linear regression*.

	Number of counties with non zero release	Univariate model		Multivariate model	
Carcinogens		Coefficient	*P* value <	Coefficient	*P* value <

Arsenic (yes : no)	2	−0.08	0.99	−2.74	0.76
Arsenic (in continuous)		−0.06	0.97	−0.50	0.73
					
1,3-butadiene (yes : no)	12	3.36	0.51	3.88	0.34
1,3-butadiene (in continuous)		0.63	0.30	0.65	0.17
					
Cadmium (yes : no)	11	5.11	0.33	2.94	0.46
Cadmium (in continuous)		1.10	0.34	0.49	0.58
					
Chromium (yes : no)	46	8.59	0.002	5.33	0.02
Chromium (in continuous)		1.43	0.0009	0.99	0.008
					
Formaldehyde (yes : no)	34	12.40	0.0001	9.08	0.0009
Formaldehyde (in continuous)		1.40	0.0001	1.02	0.001
					
Nickel (yes : no)	43	9.57	0.0008	5.98	0.01
Nickel (in continuous)		1.39	0.002	0.95	0.01
					
No release of selected chemicals (reference)	68				
Low total amount of release^†^	38	5.44	0.07	4.47	0.05
High total amount of release^†^	30	14.07	0.0001	12.34	0.0001

Total amount (in continuous)		1.28	0.0001	1.10	0.0001

*The amount of each selected chemical in continuous and total amount of all selected chemicals were log transformed. ^†^The cut point for low or high release of total amount of selected chemicals was set at 2000 pounds annually (low: <2000 pounds, high: ≥2000 pounds). In the multivariate model for each selected chemical, adjusted variables included proportion of nonwhite population in 1990, prevalence of smoking in 2003–2006, proportion of male population, proportion with college or higher education in 1990, proportion of families below poverty in 1990, and metro or nonmetro counties (metro counties: Rural-Urban Continuum Code < 4; nonmetro counties: Rural-Urban Continuum Code ≥ 4).

**Table 3:** Association of lung cancer incidence with six selected chemicals using linear regression by gender*.

Carcinogens	Males Coefficient	*P* value <	Females Coefficient	*P* value <
Arsenic (yes : no)	−5.47	0.67	−0.77	0.93
Arsenic (in continuous)	−0.95	0.65	−0.17	0.90
				
1,3-butadiene (yes : no)	3.30	0.58	5.00	0.19
1,3-butadiene (in continuous)	0.65	0.34	0.74	0.10
				
Cadmium (yes : no)	2.06	0.72	3.87	0.30
Cadmium (in continuous)	0.06	0.97	0.88	0.29
				
Chromium (yes : no)	6.42	0.06	5.20	0.02
Chromium (in continuous)	1.20	0.03	0.94	0.007
				
Formaldehyde (yes : no)	10.04	0.01	8.92	0.0005
Formaldehyde (in continuous)	1.13	0.01	1.00	0.0007
				
Nickel (yes : no)	6.92	0.04	5.90	0.007
Nickel (in continuous)	1.07	0.05	0.96	0.006
				
No release of selected chemicals (reference)				
Low total amount of release^†^	5.55	0.10	4.19	0.05
High total amount of release^†^	13.74	0.001	12.25	0.0001

Total amount (in continuous)	1.28	0.009	1.07	0.0001

*The amount of each selected chemical in continuous and total amount of all selected chemicals were log transformed. ^†^The cut point for low or high release of total amount of selected chemicals was set at 2000 pounds annually (low: <2000 pounds, high: ≥2000 pounds). In the multivariate model for each selected chemical, adjusted variables included proportion of nonwhite population in 1990, prevalence of smoking in 2003–2006, proportion of male population, proportion with college or higher education in 1990, proportion of families below poverty in 1990, and metro or nonmetro counties (metro counties: Rural-Urban Continuum Code < 4; nonmetro counties: Rural-Urban Continuum Code ≥ 4).

**Table 4:** Association of lung cancer incidence with six selected chemicals using linear regression by region*.

	Metro counties		Nonmetro counties	
Carcinogens	Coefficient	*P* value <	Coefficient	*P* value <
Arsenic (yes : no)	−4.10	0.60	—	—
Arsenic (in continuous)	−0.70	0.58	—	—
				
1,3-butadiene (yes : no)	2.33	0.62	6.16	0.35
1,3-butadiene (in continuous)	0.40	0.53	0.86	0.20
				
Cadmium (yes : no)	4.04	0.38	0.19	0.98
Cadmium (in continuous)	0.20	0.84	0.24	0.89
				
Chromium (yes : no)	3.65	0.25	6.60	0.04
Chromium (in continuous)	0.42	0.34	1.65	0.004
				
Formaldehyde (yes : no)	4.30	0.22	12.15	0.005
Formaldehyde (in continuous)	0.59	0.12	1.35	0.01
				
Nickel (yes : no)	3.48	0.27	7.49	0.02
Nickel (in continuous)	0.39	0.37	1.56	0.008
				
No release of selected chemicals (reference)				
Low total amount of release^†^	2.09	0.63	4.82	0.08
High total amount of release^†^	6.64	0.08	17.53	0.0002

Total amount (in continuous)	0.61	0.10	1.37	0.0001

*The amount of each selected chemical in continuous and total amount of all selected chemicals were log transformed. ^†^The cut point for low or high release of total amount of selected chemicals was set at 2000 pounds annually (low: <2000 pounds, high: ≥2000 pounds). Covariates included proportion of nonwhite population in 1990, prevalence of smoking in 2000, proportion of male population, proportion of people with college or higher education in 1990, and proportion of families below poverty in 1990.
